# Retraction: The anti-vaccination infodemic on social media: A behavioral analysis

**DOI:** 10.1371/journal.pone.0279796

**Published:** 2022-12-22

**Authors:** The PLOS ONE Editors

Following the publication of this article [1], concerns were raised regarding the methodology, results, and conclusions presented in this article. The editorial team and a subject expert have re-evaluated the article and determined that the concerns listed below remain unresolved.

- The sampling reported in the study is inadequate; the sample size is too small, and the study does not report adequate information to assess whether the selected sample is sufficiently representative. In addition, it appears no attempt was made to match profiles by profile characteristics such as number of followers, how long the profile has been active, or the number of tweets.
- The anti-vaccination and pro-vaccination search terms used in the study may not have been balanced appropriately and the study does not report on the justification for the choice of hashtags used. Similarly, the use of a random word generator to create a random hashtag to use as control is inappropriate and suggests that the study did not include an appropriate characterization of underlying Twitter behavior.
- The study does not provide an adequate definition of “emotional language”, and the related results reporting on the use of emotional language include an outlier data-point in the pro-vaccine group, which could drive the effect significantly in a study with a small sample size.
- The network analysis includes only a small number of profiles with an unbalanced number of neighbors. In addition, the clustering coefficient is inappropriate and the absence of confidence intervals in Fig 5C is problematic. As currently presented, these results are not sufficient to draw meaningful conclusions.
- The reported conclusion “Our data demonstrate that Donald Trump, before his profile was suspended, was the main driver of vaccine misinformation on Twitter.” is not supported by the research reported in this study. Although the reported results suggests that people who tweet anti-vaccine content are likely to be in Trump’s network, the reported results are not sufficient to support the claim that Trump himself is driving vaccine misinformation.

The authors commented that the retrieved Twitter webs and associated communities of pro- and anti-vaccine users and influencers present a snapshot taken at a given moment in time, which can be expected to change with changing opinion, circulating contents, and relevance of users within and outside of the community. Furthermore, they clarified that the study is a proxy to catch anti- and pro-vaccine discourse on Twitter and does not claim that their study is comprehensive of all vaccine-related-views on social media.

In response to the issues raised above, the authors explained that the hashtags used in the study are the most widely adopted by the pro-vaccine and anti-vaccine community, since, according to the authors, most users within the pro- and anti-vaccination communities used the chosen hashtags. The authors agreed that a different choice of hashtags would likely have led to different results but stated that these results would present a similar underlying interpretation, meaning, and value.

Regarding the sample size concerns, the authors explained that currently available software is not as able to identify misinformation with high confidence, and thus a manual assessment of each tweet needed to be performed. For this reason, the authors stated that the sample size of 50 was chosen as a trade-off between time required to conduct the analysis and the need for a meaningful and representative sample size. They stated that the sample was randomly chosen and is at least in part representative of a wider population, but they agreed that the sample may not be representative of the population at large when including people who are not active on Twitter.

Concerning the definition of “emotional language”, the authors explained that they rely on a broad definition, i.e., the use of specific words or combination of words to describe or evoke an emotional reaction. The authors further explained that they excluded outliers, as described in the methods section of the paper, since these are behavioral outliers, which indicate behaviors outside the norm. These could be due to either users with a large number of followers, which do not predict the behavior of most users on Twitter, or bots.

Furthermore, the authors comment that the unbalanced number of neighbors is a finding which explains that users in the anti-vaccine community are more engaged in discussion with one another, and users in this community tend to share content from influencers in this community, whereas the number of neighbors in the pro-vaccine community is lower because similar connections between users and similarly large influencers do not exist in this group. The authors also state that the confidence interval is missing from Fig 5C because the analysis represents a snapshot of the Twitter connections at the time of analysis, and that the data rely on an n = 1 data point. A subject expert assessed the authors’ response and commented that the difference in number of connections between groups is not a sufficient reason to not find an equivalently sized control group in the vaccine community to support the additional analyses, beyond counting neighbors of a small group of users. Furthermore, the subject expert commented that the assumption that a single clustering coefficient can only be calculated for a network is incorrect, and instead that local clustering coefficients can be calculated for each node, the average of which is the network clustering coefficient, allowing for estimation of the confidence intervals. In response to the expert’s comments, the authors stated that identifying a control web with a similar number of neighbors, as suggested by the subject expert, would neither be feasible nor reasonable, since, according to the authors, Fig 5 shows that the two Twitter webs (pro- vs anti-vaccination) are indeed different and have a different number of neighbors, which, they claim, is an important finding of their research. Regarding the confidence interval in Fig 5C, the authors claim this is not necessarily required, since the connections of the peripheral profiles in the webs have not been considered in their analysis.

Regarding the conclusion, the authors agreed that the sentence regarding Trump’s role, taken out of context, is not supported by the data presented in the paper, but they maintain that their results, as described in the discussion section, show that Trump’s profile was, at the time, “the main influencer in the anti-vaccination web” on Twitter.

The *PLOS ONE* Editors retract this article [1] because, per our editorial assessment, it did not meet *PLOS ONE*’s publication criteria (#3, 4) [2]. We regret that the issues with the article were not identified and addressed prior to its publication.

Both authors did not agree with the retraction and stand by the article’s findings.

## References

[1] Germani F, Biller-Andorno N (2021) The anti-vaccination infodemic on social media: A behavioral analysis. PLoS ONE 16(3): e0247642. 10.1371/journal.pone.0247642 33657152 PMC7928468

[2] https://journals.plos.org/plosone/s/criteria-for-publication

